# Data on three-year pesticide monitoring in ditches of the apple orchard region of Altes Land, Germany

**DOI:** 10.1016/j.dib.2018.03.074

**Published:** 2018-03-21

**Authors:** Stefan Lorenz, Gabriela Bischoff, Matthias Stähler

**Affiliations:** a Julius Kuehn Institute, Institute for Ecological Chemistry, Plant Analysis and Stored Product Protection, Koenigin-Luise-Strasse 19, 14195 Berlin, Germany; b Julius Kuehn Institute, Institute for Bee Protection, Koenigin-Luise-Strasse 19, 14195 Berlin, Germany

## Abstract

The data presented in this article are related to the research article 'Chemical and biological monitoring of the load of plant protection products and of zoocoenoses in ditches of the orchard region Altes Land' (Süß et al., 2006) [1], which is only available in the German language. The pesticide data presented here were acquired from four ditches (three ditches were located in apple orchards, and one ditch was located in a grassland region) between 2001 and 2003 (Lorenz et al., 2018) [2]. Two different monitoring strategies were applied: event-driven sampling after pesticide applications and weekly integrated sampling using automatic water samplers. A total of 70 active substances were monitored while farmers applied 25 active substances. This article describes the study sites and the analytical methods used to quantify the pesticides in the water samples. The field data set is publicly available at the OpenAgrar repository under https://doi.org/10.5073/20180213-144359 (Lorenz et al., 2018) [2].

## Specifications Table

**Table:** 

Subject area	*Environmental science*
More specific subject area	*Freshwater ecology, ecotoxicology, environmental monitoring*
Type of data	*Table*
How data were acquired	*GC–MS and/or LC-MS/MS measurements following solid-phase extraction of event-driven and weekly integrated water sampling*
Data format	*Filtered data (means of duplicate measurements)*
Experimental factors	*None*
Experimental features	*None*
Data source location	*Orchard region of Altes Land, Germany; ditches in Neuenkirchen, Jork and Estebrügge (2 ditches)*
Data accessibility	*The field data set is publicly available at the OpenAgrar repository under*https://doi.org/10.5073/20180213-144359
	https://www.openagrar.de/receive/openagrar_mods_00036710

## Value of the data

• Pesticide contamination monitoring of small water bodies is extremely challenging as spatial and temporal factors affect the maximum peak concentrations of pesticides.
• The event-driven and weekly integrated data presented here allow for realistic assessment of potential exceedances of environmental quality standards following pesticide application.
• The data allow other researchers to perform statistical (meta-)analysis on the impact of agriculture on freshwater ecosystems.

## 1 Data

The data presented in this article consist of pesticide concentration data from event-driven and weekly integrated monitoring of 70 active substances in four ditches of the orchard region of Altes Land, Germany [1, 2]. Three ditches were located in an apple orchard, and one ditch was located in a grassland region. All apple farmers in the orchard region provided their data on pesticide application from 2001 to 2003. A total of 493 pesticide applications (mostly on both sides of the ditches), including 27 different plant protection products containing 25 active substances, were recorded (Table 1). Applications of biological plant protection products were not recorded. All water samples were analysed using a multi-method design validated for 70 active compounds (26 fungicides, 23 herbicides, 13 insecticides, 2 acaricides and 6 metabolites). The list of analytes is given in Table 2. The data consist of one Excel sheet providing the results of the pesticide analysis, surrogate recovery, and description of the sampling details (study site, sample type, sampling date, applied pesticides and pH), which all refer to a unique sample identifier. The field data set is publicly available at the OpenAgrar repository under https://doi.org/10.5073/20180213-144359 [2].

**Table 1:** List of pesticide active substances applied by the farmers with the number of applications from 2001 to 2003. Italic substances denote compounds showing simultaneous insecticide/acaricide efficacy.

	**Year**				**Year**		
**Fungicides**	2001	2002	2003	**Insecticides**	2001	2002	2003
benomyl	3	3		beta-cyfluthrin	4		4
captan	34	39	43	fenoxycarb			1
copper	31	16	12	*fenpyroximate*	2		2
cyprodinil		1	3	indoxacarb			2
dichlofluanid	7	4	7	methoxyfenozide			1
dithianon	4	1	5	*oxydemeton-methyl*	3	4	
fluquinconazole	6	4	6	pirimicarb		5	1
kresoxim-methyl	2	2		tebufenozide	1	1	1
metiram	10	4	7	thiacloprid			3
penconazole	3	4	8				
pyrimethanil	7	4	6	**Growth regulators**			
triadimenol	3			naphthylacetamide	2		
trifloxystrobin		1					
sulphur	44	68	52	**Herbicides**			
				glyphosate	2

**Table 2:** List of pesticide active substances included in the ditch monitoring. Italic substances denote compounds showing simultaneous insecticide/acaricide efficacy.

**Herbicides**	**Fungicides**	**Insecticides**
atrazine	azoxystrobin	azinphos-methyl
bifenox	captan	beta-cyfluthrin
carbetamide	carbendazim^a^	fenoxycarb^f^
chloridazon	cis-1,2,3,6-tetrahydrophthalimide^b^	*fenpyroximate*
chlortoluron	copper	imidacloprid
cyanazine	cyproconazole	indoxacarb^f^
desethyl-atrazine	cyprodinil	lambda-cyhalothrin
(DE-atrazine)	dichlofluanid	methoxyfenozide^f^
desethyl-terbuthylazine	difenoconazole	*oxydemeton-methyl*
(DE- terbuthylazine)	N,N-dimethylsulfamide	parathion
desisopropyl-atrazine	(DMSA = N,N-DMS)^c^	pirimicarb
(DIP-atrazine)	N,N-dimethyl-N’-p-tolyl-sulfamide	tebufenozide
diflufenican	(DMST)^d^	thiacloprid^f^
dimefuron	epoxiconazole	
diuron	fenpropidin	
ethofumesate	fenpropimorph	**Acaricides**
flufenacet	fluquinconazole	clofentezine
isoproturon	kresoxim-methyl	tetradifon
linuron	metconazole	
metamitron	myclobutanil	
metazachlor	penconazole	
metobromuron	prochloraz	
metolachlor	propiconazole	
metoxuron	pyrimethanil	
metribuzin	quinoxyfen	
propaquizafop	spiroxamine	
propazine	tebuconazole	
simazine	tolylfluanid	
terbuthylazine	triadimenol	
	trifloxystrobin^e^	
	vinclozolin	

a can be a metabolite of benomyl.

b metabolite of captan used for the detection of captan-containing pesticides as captan itself shows extremely short half-life periods.

c metabolite of dichlofluanid.

d metabolite of tolylfluanid.

e analyzed in 2002–2003.

f only analyzed in 2003.

## 2 Experimental design, materials and methods

### 2.1 Study site description

The data were gathered in four representative ditches of the orchard region of Altes Land, Germany, which is close to the Lower Elbe region near the city of Hamburg. The characteristics of all four ditches are presented in Table 3. The selection criteria for the three ditches located directly within the apple orchard were as follows: (i) similarity in hydromorphology and macrophyte cover combined with (ii) distinct differences in pesticide exposure risk due to different management measures and distances from water sources. The fourth ditch was selected as an additional reference site from 2002 on as this ditch was located in a grassland region without pesticide application. All ditches were permanently water-bearing with a negligible current.

**Table 3:** Site characteristics of the four studied ditches. Values are given as mean with minimum/maximum range in brackets. IPP = integrated plant protection, DOC = dissolved organic carbon.

	**Study site**			
	Neuenkirchen	Estebrügge	Jork	Estebrügge
culture	apple	apple	apple	grassland
farming	IPP	IPP	organic	–
water/orchard distance	2.9	4.5	5.8	> 50
bank/orchard distance	2.4	3.8	3.8	> 50
width [m]	3.4	4.0	2.9	3.0
depth [cm]	53 (36–70)	49 (20–71)	56 (40–89)	29 (10–47)
water temperature [°C]	18 (6–26)	17 (8–22)	17 (8–24)	17 (8–23)
pH	7.3 (6.5–9.2)	7.4 (6.4–9.2)	7.6 (6.9–9.5)	7.2 (7.0–8.1)
dissolved oxygen [mg/L]	9.0 (3.2–14.3)	7.0 (1.1–17.8)	8.4 (2.7–16.9)	5.9 (0.8–11.3)
dissolved oxygen [%]	93 (31–149)	70 (11–170)	87 (29–161)	61 (8–112)
conductivity [µS]	361 (185–513)	914 (334–1378)	523 (312–939)	1101 (486–1750)
NH_4_^+^ [mg/L]	0.85 (0.3–1.7)	0.91 (0.2–2.2)	0.93 (0.2–2.1)	1.24 (0.3–2.3)
PO_4_^3−^ [mg/L]	0.12 (0.01–0.49)	0.75 (0.01–2.1)	0.31 (0.01–1.5)	0.38 (0.03–0.78)
hardness [°dH]	8 (3–10)	12 (6–18)	12 (6–17)	9 (4–11)
DOC [mg C/L]	24 (15–35)	20 (12–33)	18 (10–32	39 (24–57)
no. of macrophyte species	27	22	22	20

Macrophyte cutting was last conducted in 1993 (Neuenkirchen ditch) and 1999 (Estebrügge and Jork ditches), prior to the monitoring. Due to the short distance between the water body and the first apple tree line of the Neuenkirchen orchard, the use of pesticide application machines between the ditch and first tree line was prevented at this site, leading to pesticide application of the first tree line directly in the path to the water body (the pesticide application machines were operated between the first and second tree lines).

### 2.2 Sample collection

Water samples were taken from April to October over three years (2001–2003). Event-driven water samples were collected manually as soon as possible after pesticide application to measure the peak pesticide concentrations. Five samples of 0.5 L were taken from the upper 20 cm of the water layer at evenly dispersed intervals along a stretch of 100 m and combined to make a 2.5-L water sample. All samples were stored in glass flasks, except for samples collected after the application of copper-bearing products, which were stored in polyethylene flasks. Weekly water samples were collected during periods without pesticide application to assess the background contamination of pesticide residues. In parallel, 30-mL samples were taken automatically at hourly intervals, providing the basis for the weekly integrated samples (36 samples in total). Samples were stored in the dark at 4 °C and analysed as soon as possible.

### 2.3 Sample analysis – all substances except copper and beta-cyfluthrin

Water samples were filtered (4–7 µm folded filter), and a surrogate standard was added (acetochlor for liquid chromatography (LC) analysis and tetrachlorvinphos for gas chromatography (GC) analysis). The surrogate standard was used to check the analytical measurement procedures and to evaluate the results, but not for result corrections. The samples were then subjected to solid-phase extraction (Chromabond HR-P cartridges, elution with methanol/ethyl acetate, 1:1, v:v). Subsequently, the samples were evaporated to dryness under a nitrogen atmosphere and dissolved in acetonitrile and an internal standard solution using an ultrasonic device [3].

LC and GC coupled to mass spectrometry – LC-MS/MS (PE Sciex API 2000) and GC–MS (Finnigan GCQ) – were used for identification and quantification of the target substances in the samples. The pesticides were identified by their retention time and multiple reaction monitoring transitions (LC-MS/MS) or full-scan spectra (GC–MS), respectively. The pesticides were quantified with reference standards in the solvent, and quantification followed the internal standard method. All results are presented as averages of duplicate injections of the sample extracts. The pesticide data were not corrected by the respective recovery rates. Fenoxycarb, indoxacarb, methoxyfenozide and thiacloprid were only analysed in 2003. Benomyl, dithianon, glyphosate, metiram, naphthylacetamide and sulphur were not analysed. The following 19 substances were analysed but not detected at levels above the limit of detection (LOD): azoxystrobin, azinphos-methyl, cyanazine, dimefuron, dichlofluanid, epoxiconazole, flufenacet, linuron, metazachlor, metobromuron, metconazole, metoxuron, propaquizafop, prochloraz, quinoxyfen, spiroxamine, tetradifon, tolylfluanid and vinclozolin.

### 2.4 Separate sample analysis for copper and beta-cyfluthrin

Copper and the pyrethroid insecticide beta-cyfluthrin were analysed separately with methods suitable for these active substances. Beta-cyfluthrin was analysed using GC coupled with an electron capture detector and GC–MS/MS following immediate filtration after sampling. The recovery rate for beta-cyfluthrin, analysed separately, was 83%, on average (relative standard deviation of 13%).

Copper residues were analysed using inductively coupled plasma optical emission spectrometry in 2001 and 2002. Therefore, the water samples were filtered through a 0.45-µm cellulose acetate membrane filter and acidified. The LOD for this method was 20 µg/L. Starting in 2003, the water samples were analysed for copper residues using atomic adsorption spectrometry to lower the LOD with this method to 0.3 µg/L.

### 2.5 Method validation and recovery testing

Method validation was performed for all substances (except for beta-cyfluthrin and copper) according to European Union Commission Directive 96/46/EC [4]. The multi-method design was validated based on recoveries from fortification experiments and periodically checked during the investigation period. Therefore, control water samples (surface water collected from the Lamspringe stream, Lower Saxony, Germany) were spiked with different concentrations of the substances (between 0.05 and 5 µg/L). The water samples were extracted using the same method as described for the samples from the ditches.

The mean recoveries of the active substances found in the water samples ranged from 70% to 110% with relative standard deviations < 10%, meeting the requirements of European Union Commission Directive 96/46/EC [4]. The LOD was 0.01 µg/L for all active substances except for dichlofluanid, methoxyfenozide and thiacloprid. The LOD for methoxyfenozide and thiacloprid was 0.05 µg/L, and the LOD for dichlofluanid was 0.1 µg/L. The limit of quantification (LOQ) was 0.05 µg/L for all active substances except for dichlofluanid, methoxyfenozide and thiacloprid. The LOQ for methoxyfenozide and thiacloprid was 0.1 µg/L, and the LOQ for dichlofluanid was 0.5 µg/L.

The recovery rate of the surrogate standards was 93%, on average (relative standard deviation of 13%), meeting the requirements of European Union Commission Directive 96/46/EC [4] for the majority of the samples analysed. The samples in the data set not meeting the criteria for surrogate recovery (range of 70–110%, [4]) should only be considered to a limited extent or not at all. Nevertheless, we decided to present the results not meeting the requirements in our data compilation. None of these respective samples, however, was taken immediately after a pesticide application event. Additionally, the surrogate standards were not added for the analysis of lambda-cyhalothrin and beta-cyfluthrin in four water samples. For these five data points (0.03% of the data), “no surrogate added” is indicated in the surrogate recovery column of the data set. Likewise, surrogate standards were not added for the analysis of copper in the water samples, and these measurements are indicated in the same manner.

### 2.6 Storage stability

Storage stability tests were performed for all substances (except for beta-cyfluthrin and copper) using surface water from the control Estebrügge ditch (grassland), according to European Union Commission Directive 96/46/EC [4]. The storage periods were selected according to the actual transport and storage conditions during the monitoring (14 days and 42 days, respectively). Storage stability was considered to be assured for a given period of time if the active substance content in the stored sample was still at least 70% compared to an immediately analysed sample [5]. The herbicides and fungicides (except for dichlofluanid) proved to be sufficiently stable over the storage periods used in this study. Among the insecticides, fenoxycarb proved to be particularly unstable. After 14 days of storage, only 10% of its initial concentration was detected. We decided to present the results of diclofluanid and fenoxycarb in the data compilation. However, please note that these data may be biased by potential underestimation.
